# Effects of intensive arm training with the rehabilitation robot ARMin II in chronic stroke patients: four single-cases

**DOI:** 10.1186/1743-0003-6-46

**Published:** 2009-12-17

**Authors:** Patricia Staubli, Tobias Nef, Verena Klamroth-Marganska, Robert Riener

**Affiliations:** 1 Sensory-Motor Systems Lab, Institute of Robotics and Intelligent Systems, ETH Zurich, Switzerland; 2 Spinal Cord Injury Center, Balgrist University Hospital, University Zurich, Switzerland; 3 Department of Biology, Institute of Human Movement Sciences and Sport, ETH Zurich, Switzerland; 4 Department of Biomedical Engineering, The Catholic University of America, Washington D.C., USA; 5 Center for Applied Biomechanics and Rehabilitation Research, National Rehabilitation Hospital, Washington D.C., USA

## Abstract

**Background:**

Robot-assisted therapy offers a promising approach to neurorehabilitation, particularly for severely to moderately impaired stroke patients. The objective of this study was to investigate the effects of intensive arm training on motor performance in four chronic stroke patients using the robot ARMin II.

**Methods:**

ARMin II is an exoskeleton robot with six degrees of freedom (DOF) moving shoulder, elbow and wrist joints. Four volunteers with chronic (≥ 12 months post-stroke) left side hemi-paresis and different levels of motor severity were enrolled in the study. They received robot-assisted therapy over a period of eight weeks, three to four therapy sessions per week, each session of one hour.

Patients 1 and 4 had four one-hour training sessions per week and patients 2 and 3 had three one-hour training sessions per week. Primary outcome variable was the Fugl-Meyer Score of the upper extremity Assessment (FMA), secondary outcomes were the Wolf Motor Function Test (WMFT), the Catherine Bergego Scale (CBS), the Maximal Voluntary Torques (MVTs) and a questionnaire about ADL-tasks, progress, changes, motivation etc.

**Results:**

Three out of four patients showed significant improvements (p < 0.05) in the main outcome. The improvements in the FMA scores were aligned with the objective results of MVTs. Most improvements were maintained or even increased from discharge to the six-month follow-up.

**Conclusion:**

Data clearly indicate that intensive arm therapy with the robot ARMin II can significantly improve motor function of the paretic arm in some stroke patients, even those in a chronic state. The findings of the study provide a basis for a subsequent controlled randomized clinical trial.

## Background

Stroke remains the leading cause of permanent disability. Recent studies estimate that it affects more than 1 million people in the EU [1,2] and more than 0.7 million in the U.S. each year [3]. The major symptom of stroke is severe sensory and motor hemiparesis of the contralesional side of the body [4]. The degree of recovery highly depends on the severity and the location of the lesion [5]. However, only 18% of stroke survivors regain full motor function after six months [6]. Restoration of arm and hand functions is essential [6] to cope with tasks of daily living and regain independence in life.

There is evidence that the rehabilitation plateau can be prolonged beyond six months post-stroke and that improvements in motor functions can be achieved even in a chronic stage with appropriate therapy [7,8]. For this to occur, effective therapy must comprise key factors containing repetitive, functional, and task-specific exercises performed with high intensity and duration [9-12]. Enhancing patients' motivation, cooperation, and satisfaction can reinforce successful therapy [13]. Robot-assisted training can provide such key elements for inducing long-term brain plasticity and effective recovery [14-19].

Robotic devices can objectively and quantitatively monitor patients' progress - an additional benefit since clinical assessments are often subjective and suffer from reliability issues [20]. Patient-cooperative control algorithms [21,22] can support patients' efforts only as much as needed, thus allowing for intensive robotic intervention.

Several clinical studies have been successfully conducted with endeffector based robots [14,16,17,23]. In these robots, the human arm is connected to the robot at a single (distal) limb only. Consequently, endeffector based robots are easy to use but do not allow single joint torque control over large ranges of motion. In general, they provide less guidance and support than exoskeleton robots [24]. In this study we propose using an exoskeleton-type robot for the intervention. Such a type of robot provides superior guidance and permits individual joint torque control [24]. The device used here is called ARMin and has been developed over the last six years [21,25].

A first pilot study with three chronic stroke patients showed significant improvements in motor functions with intensive training using the first prototype ARMin I. Since ARMin I provided therapy only to the shoulder and elbow, there were no improvements in distal arm functions [25]. Consequently, the goal was to develop a robot, which enables a larger variability of different (also more complex and functional) training modalities involving proximal and distal joint axes [26,27].

**Figure 1:**
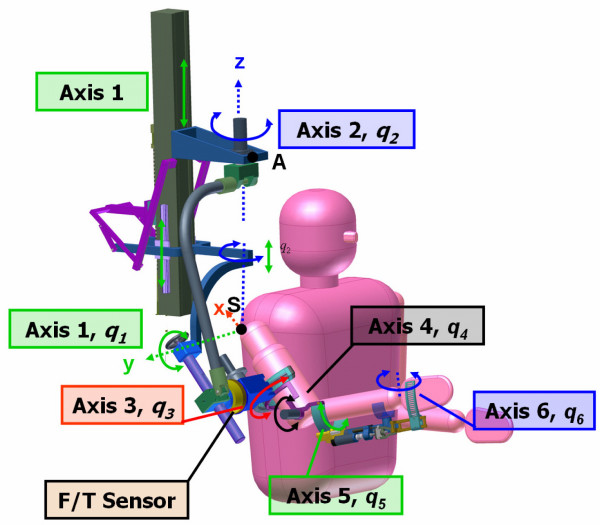
**Mechanical structure of the exoskeleton robot ARMin II**. Axis 1: Vertical shoulder rotation, Axis 2: Horizontal shoulder rotation, Axis 3: Internal/external shoulder rotation, Axis 4: Elbow flexion/extension, Axis 5: Pro/supination of the lower arm, Axis 6: Wrist flexion/extension.

For this study we used an enhanced prototype, ARMin II, with six independently actuated degrees of freedom (DOF) and one coupled DOF (Figure 1). The robot trains both proximal joints (horizontal and vertical shoulder rotation, arm inner - outer rotation, and elbow flexion - extension) and distal joints (pro - supination of lower arm and wrist flexion - extension). Together with an audiovisual display, ARMin II provides a wide variety of training modes with complex exercises and the possibility of performing motivating games.

The goal of this study was to investigate the effects of ARMin II training on motor function, strength and use in everyday life.

## Methods

### Participants

Four patients (three male, one female) met the inclusion criteria and volunteered in the study. The inclusion criteria were i) diagnosis of a single ischemic stroke on the right brain hemisphere with impairment of the left upper extremity and ii) that stroke occurred at least twelve months before study entrance.

Study exclusion criteria were 1) pain in the upper limb, so that the study protocol could not be followed, 2) mental illness or insufficient cognitive or language abilities to understand and follow instructions, 3) cardiac pacemaker, and 4) body weight greater than 120 kg.

All four patients received written and verbal information about the study and gave written informed consent. The protocol of the study was approved by the local ethics committee.

### Procedure

To investigate the effects of training with the rehabilitation device ARMin II, four single-case studies with A-B design were applied. Clinical evaluations of the Fugl-Meyer Score of the upper extremity Assessment (FMA), the Wolf Motor Function Test (WMFT), the Catherine Bergego Scale (CBS), and the Maximal Voluntary Torques (MVTs) were administered twice during a baseline period of three weeks (A). A training phase of eight weeks (B) followed. The same evaluation tools were applied every two weeks. Patients 1 and 4 executed three training hours per week (totally 24 hours over entire training period), patients 2 and 3 completed four training hours per week (totally 32 hours). A single training session comprised approximately 15 minutes passive mobilization and approximately 45 minutes active training. Training sessions were always led by the same therapist.

### Robotic therapy

ARMin II [21] allows for complex proximal and distal motions in the functional 3-D workspace of the human arm (Figure 1). The patient sits in a wheelchair (wheels locked) and the arm is placed into an orthotic shell, which is fixed and connected by three cuffs to the exoskeletal structure of the robot. Position and force sensors support active and passive control modes. Two types of therapy modes were applied: a passive 'teach and repeat' mobilizing mode and a game mode with active training modalities.

For the passive therapy, the therapist can carry out a patient-specific mobilization sequence adapted to individual needs and deficits, using the robot's 'teach and repeat' mode. The therapist guides the mobilization ('teach') by moving the patient's arm in the orthotic shell. The trajectory of this guided mobilization is recorded by the robot, so that the same mobilization can be repeated several times ('repeat'). The patient receives visual feedback from an avatar on the screen, that performs the same movements in real-time. During the teaching sessions, the robot is controlled by a zero-impedance mode, in which the robot does not add any resistance to the movement, so that the therapist consequently only feels the resistance of the human arm. During the 'repeat' mode, the robot is position-controlled and repeats the motion that has been recorded before.

**Figure 2:**
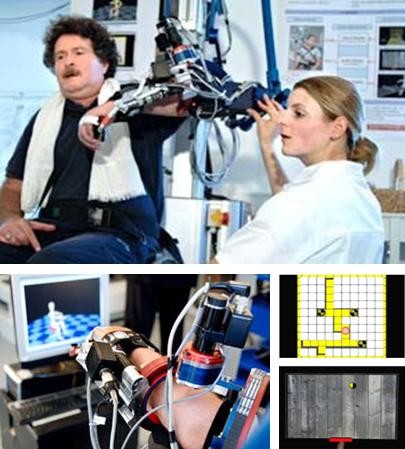
**Subject in the robot ARMin II with labyrinth and ball game scenario**.

For the active part of the therapy, a ball game and a labyrinth scenario were selected (see Figure 2). In the ball game, the patient moves a virtual handle on the screen. The aim is to catch a ball that is rolling down a virtual ramp by shifting the handle. When a patient is unable to succeed, the robot provides support by directing the handle to the ball (ARMin II in impedance-control mode). To give the patient visual feedback, the color of the handle turns from green to red when robot-support is delivered. Acoustic feedback is provided when a ball is precisely caught. The difficulty level of the ball game can be modified and adjusted to the patient's need by the therapist, i.e. the number of joint axes involved, the starting arm position, the range of motion, the robotic assistance, resistance or opposing force, and speed.

In the labyrinth game, a red ball (cursor) moves according to the patient's arm motions. The objective is to direct the ball from the bottom to the top of the labyrinth. The cursor must be moved accurately. If the ball touches the wall too hard, it drops to the bottom and the game restarts. Like the ball game, the labyrinth provides various training modalities by changing the settings, such as the amount of arm weight compensation, vertical support, number of joint axes involved, working space and sensitivity of the wall [28].

### Outcome measurements

To ensure reproducibility and consistency of the testing procedure, all measurements were executed by the same person and with the same settings for each patient. Evaluations were always completed before training sessions. Clinical assessments were filmed and later evaluated by an independent "blinded" therapist from "Charité, Median Clinic Berlin, Department Neurological Rehabilitation".

The main clinical outcome was the Fugl-Meyer Assessment (FMA) of the upper-limb. This impairment-based test consists of 33 items with a total maximum score of 66. The test records the degree of motor deficits and reflexes, the ability to perform isolated movements at each joint and the influence of abnormal synergies on motion [29]. It shows good quality factors (reliability and validity) [30,31] and it is widely used for clinical and research assessments [32].

The Wolf Motor Function Test (WMFT) is a 15-item instrument to quantify disability and to assess performance of simple and complex movements as well as functional tasks [33]. This test has high interrater reliability, internal consistency, and test-retest reliability [34]. The WMFT is responsive to patients with mild to moderate stroke impairments. However, for severely affected patients it has low sensitivity due to a floor effect (when single test items are too difficult).

Severity of neglect was evaluated with the Catherine Bergego Scale (CBS), a test that shows good reliability, validity [35], and sensitivity [36].

To assess sensory functions of the upper limb, the American Spinal Injury Association (ASIA) scoring system was used [37]. The degree of sensation to pinprick (absent = 0, impaired = 1, normal = 2) was determined at the key sensory points of the C4 to T1 dermatomes. The single scores were summed.

In addition, a questionnaire was designed, referring to ADL-tasks, progress, changes, motivation etc. The patients then had to rate the different questions on a scale from 1 to 10, and furthermore, add a comment, expressing their subjective experiences and impressions.

### Measurements with ARMin II

With the ARMin II robot, maximal voluntary torques (MVTs) were determined for six isometric joint actions including vertical shoulder flexion and extension, horizontal shoulder abduction and adduction, as well as elbow flexion and extension. Patients were seated in a locked wheelchair with the upper body fixed by three belts (two crosswise diagonal torso belts and one belt over the waist) to prevent the torso from assisting the movements. The starting position was always the same. The shoulder was flexed 70° and transversally abducted 20°, the rotation of the upper and lower arm was neutral (0°), and the elbow was flexed 90°. Patients were instructed to generate maximal isometric muscle contractions against the resistance of ARMin II for at least two seconds before relaxing. During the effort, verbal encouragement was given in each case.

### Data analysis

From the main baseline measurements - FMA, WMFT, CBS, and MVT - the mean values and standard deviations were calculated. Data recorded during the intervention phases were evaluated by using the least square linear regression model with applied bootstrap resampling technique [38]. For the statistical analysis, the programs SYSTAT 12 and Matlab 6.1 were used. The significance level p ≤ 0.05 of the slope of the regression line was considered to indicate a statistically significant improvement.

## Results

**Figure 3:**
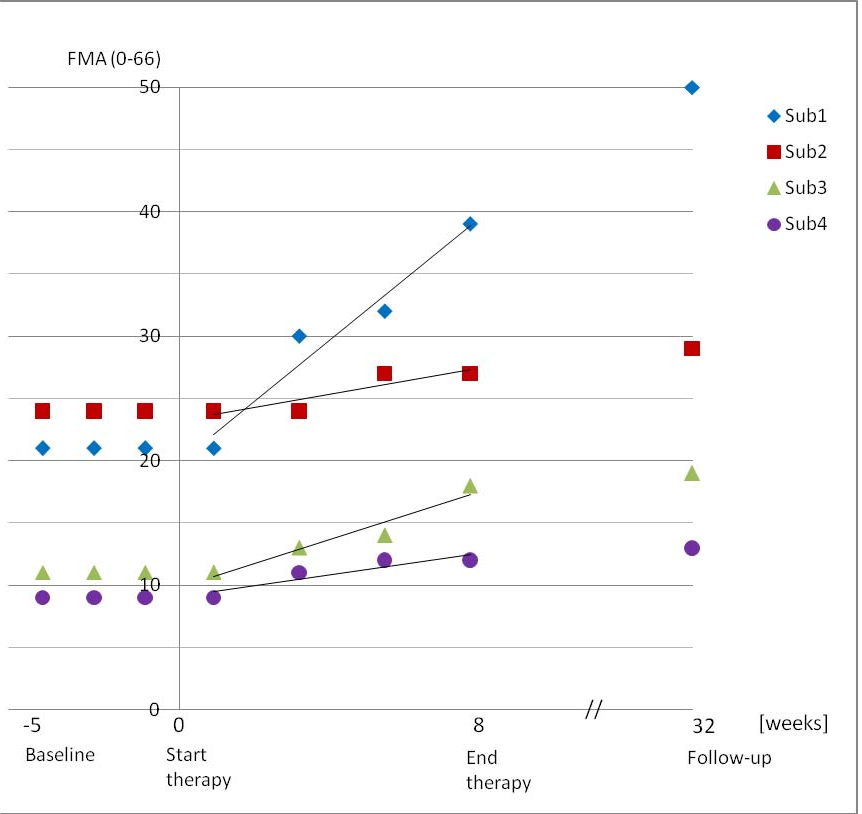
**Clinical FMA scores across evaluation sessions**.

The results of the FMA are presented in Table 1. From baseline to discharge, patients 1, 2, and 3 increased their scores significantly (p < 0.05). They continued to improve in the FMA at the six-month follow-up (see Figure 3).

**Table 1:** Overview of the Fugl-Meyer Assessment

FMA: Total^§^ sh/e^§^ w/h^§^	Baseline	Post- therapy	Difference^†^	Follow up (6 mt)	Difference^‡^	Total change	R^2^	p
S1: Total	21	38.6	+17.6	50	+11.4	+29	0.943	0.001*
sh/e	20	24.0	+4.0	28	+4.0	+8		
w/h	1	14.6	+13.6	22	+7.4	+21		
								
S2: Total	24	27.1	+3.1	29	+1.9	+5	0.800	0.041*
sh/e	21	23.1	+2.1	24	+0.9	+3		
w/h	3	4.0	+1.0	5	+1.0	+2		
								
S3: Total	11	17.8	+6.8	19	+1.2	+8	0.908	0.003*
sh/e	10	15.8	+ 5.8	18	+2.2	+8		
w/h	1	2.0	+1.0	1	-1.0	+0		
								
S4: Total	10	12.1	+2.1	13	+0.9	+3	0.408	0.172
sh/e	10	12.1	+2.1	12	-0.1	+2		
w/h	0	0	+0	1	+1.0	+1		

Note: An increase in score indicates improvement. S1 - S4 means subject 1 to 4.

§Fugl-Meyer (FMA), total score, maximum = 66; score for shoulder/elbow (sh/e), max. = 36; score for wrist/hand (w/h), max. = 30

†Difference of score between baseline and post-test.

‡Difference of score between post-test and follow up.

*Indicate significant p-values < 0.05

Patient 1 gained +17.6 points in the FMA (from 21 to 38.6 points), while at the follow-up, six months later, he demonstrated even further impressive progress, without having received additional therapy in the mean time. Overall, patient 1 showed an absolute improvement of +29 points (from 21 to 50 points), particularly due to high recovery in distal arm functions (+21 points).

The FMA gains of patients 2 and 3 were +5 points (from 24 to 29 points) and +8 points (from 11 to 19 points). These findings were in line with other investigations about the effects of robot-assisted therapy in chronic stroke patients that demonstrated changes between 3.2 and 6.8 points [14,23,39-43]. However, one must note that such comparisons have to be done with care since studies often differ in methods and criteria (e.g. intervention time, number of training sessions per week, duration of training sessions, type of stroke, affected brain side, time post-stroke, and severity of lesion). Patient 4 showed an increase of +3 points (from 10 to 13 points) in the FMA; however, this increase was statistically not significant.

**Figure 4:**
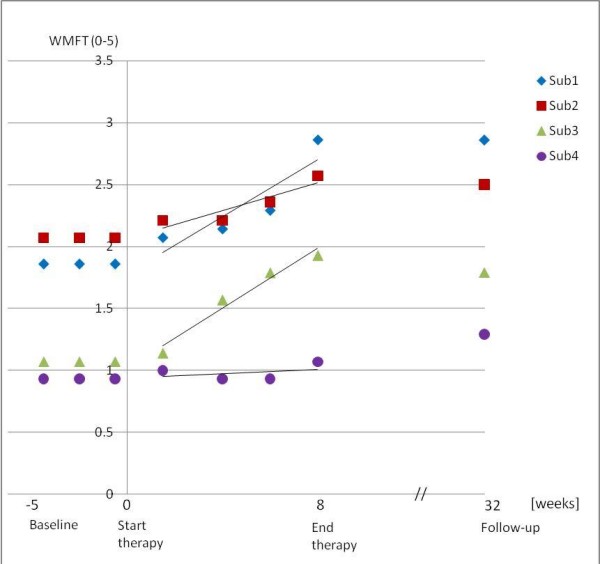
**Clinical WMFT scores across evaluation sessions**.

Typical arm functions that are relevant for activities of daily life can be expressed by the WMFT (Table 2). During the therapy, the WMFT scores of patients 1, 2 and 3 increased by +1.00, +0.5, and +0.86 points, respectively. Patients 2 and 3 slightly diminished at follow-up. Nevertheless, these three patients achieved significant progress (p < 0.05), in contrast to patient 4, who showed no significant improvement. However, at the follow-up examination, patient 4 was the only one who further improved in the WMFT (see Figure 4).

**Table 2:** Overview of the Wolf Motor Function Test

WMFT^§^	Baseline	Post- therapy	Difference^†^	Follow up (6 mt)	Difference^‡^	Total change	R^2^	p
S1:	1.86	2.86	+1.00	2.86	0	+1.00	0.911	0.003*
S2:	2.07	2.57	+0.50	2.50	-0.07	+0.43	0.891	0.005*
S3:	1.07	1.93	+0.86	1.79	-0.14	+0.72	0.831	0.011*
S4:	0.93	1.07	+0.14	1.29	+0.22	+0.36	0.577	0.080

Note: An increase in score indicates functional improvement. S1 - S4 means subject 1 to 4.

§Wolf Motor Function Test (WMFT), 5 = normal motor functions.

*Indicate significant p-values < 0.05

A questionnaire was used to obtain further information about patient status. The patients reported progress of the affected upper extremity in everyday life activities (e.g. the arm can be lifted higher and better, is more integrated, feels lighter and is less stiff, able to lift glass, fold laundry, use index finger, and control motions better). The grades of patients 1 to 4 regarding the use of their impaired arm during ADLs after the intervention (scale range 1 to 10, no better use = 1, much better use = 10) were 5, 7, 4 and 3, respectively. Furthermore, they described to be more motivated and willing to try to engage their arm in diverse daily activities.

An overview of the MVTs, consisting of six different torque measurements, is presented in Table 3. At the follow-up, improvement in muscle strength increased in patient 1, while it slightly diminished in patients 2 and 3. In patient 4, muscle strength returned to the base level at the follow-up.

**Table 3:** Overview of Total Maximal Voluntary Torques (MVTs)

MVT^§ ^(Nm)	Baseline	Post- therapy	Difference^†^	Follow up (6 mt)	Difference^‡^	Total change
S1:	17.6 ± 7.5	31.6 ± 9.0	+14.0	36.4 ± 10.9	+4.8	+18.8
S2:	6.1 ± 5.2	14.4 ± 3.4	+8.3	12.9 ± 5.2	-1.5	+6.8
S3:	9.6 ± 6.7	22.4 ± 10.7	+12.7	19.2 ± 13.3	-3.2	+9.6
S4:	2.6 ± 2.0	8.3 ± 3.4	+5.7	3.4 ± 2.9	-4.9	+0.8

Note: An increase in Nm indicates improvement in muscle torque. S1 - S4 means subject 1 to 4.

§Total of maximal voluntary torques (Nm): Consists of 6 different torque measurements (shoulder flexion - extension; horizontal shoulder rotation; elbow flexion - extension).

†Difference of Nm between baseline and post-test.

‡Difference of Nm between post-test and follow up.

The demographic data and clinical characteristics of the four patients are summarized in Table 4. None of the patients reported any adverse effects from robot-mediated therapy. In contrast, patients 3 and 4 described reduced hardening and pain of their neck and shoulder muscles.

**Table 4:** Data on the Subjects at Admission

	S1	S2	S3	S4
Gender	Male	female	male	male
Age	39	60	54	58
Handedness (before stroke)	Right	right	right	right
Hemisphere of unilateral stroke	Right	right	right	right
Diagnosis of stroke	ischemic media insult right, bleeding into Ncl. Lentiformis	ischemic media insult right, in the temporal dorsal brain	ischemic insult in the right PCA*	ischemic media insult right
Months post-stroke (at entrance)	12	131	22	16
Reflex Status (0/+/++/+++)	++	+++	+++	+++
Sensation, pin prick, C4-T1 (0-24)	20	22	24	7
F. Independence Measure (18-126)	103	121	112	90
Fugl-Meyer Assessment UL (0-66)	21	24	11	9
Wolf Motor Function Test (0-5)	1.80	2.07	1.01	0.35
Catherine Bergego Scale (0-30)	5	4	12	16
Modified Asworth Scale (0-5)				
Elbow	0	3	3	3
Wrist	0	3	3	3
Finger	2	3	3	3

Note: Tests refer to the impaired body side. Functional Independence Measure: 18 = being completely dependent, 126 = acting completely independent. Wolf Motor Function Test: 0 = no motor functions, 75 = normal motor functions. Fugl-Meyer Assessment: 0 = no motor functions, 66 = normal motor functions. Catherine Bergego Scale: Neglect: 0 = normal, 30 = severe neglect. Modified Asworth: 0 =no spasticity, 5 = severe spasticity. Reflex Status: 0 = no reflex, '+' = moderate reflex,

'++' = normal reflex, '+++' = vigorous reflex. Sensation, pin prick C4 to T1: absent = 0, impaired = 1, normal = 2, max 24.

Patients 1, 2 and 3 completed measurements and therapy sessions, except for patient 4, who missed one measurement date and two therapy sessions for reasons that are not related to the study.

## Discussion

In this study, intensive therapy using the robot ARMin II was administered to four chronic stroke patients during eight weeks of training. Patients 1 and 4 received 32 and 30 hours of therapy respectively, while patients 2 and 3 received 24 hours.

The results of these single-case series underline prior findings with robotic therapy, namely that intensive, repetitive, task-specific, and goal-directed training can significantly improve motor functions in chronic stroke patients - even years post-stroke [14,23,44]. All four patients demonstrated improvements in motor and functional activities, but to various degrees. Overall, they sustained their functional gains at the six-month follow-up or even continued to improve after the end of treatment, indicating potential long-term benefits of robot-assisted therapy.

Patient 4 had the lowest motor functions at study entrance and hardly any sensation in the clinical pinprick test. Such neurological deficits can make functional therapy very difficult as feedback functions are not, or hardly, available. This might explain why patient 4 could only profit little from the training with ARMin II. For stroke individuals with little sensory functions, as e.g. patient 4, a sensory intervention is suggested to be a more effective approach [45]. In general, it can be said that stroke patients with severe sensory loss benefited less from treatment than moderately impaired patients [10,46].

The gains in the WMFT likely reflect increased motor performance levels that are suggested to facilitate use of the impaired upper limb in daily activities. These changes seemed to be clinically significant from the patients' perspective. However, the analysis suggested that the impaired upper limb was mainly involved as an assist in bimanual ADLs after intervention. In addition, one must note that not only gains in motor abilities were achieved, but also positive impacts on concentration, neglect, physical capacity, well-being, body balance and posture were noticed. Patient 4, for example, diminished twelve points in the CBS, indicating a reduced neglect (Table 5).

**Table 5:** Overview of Catherine Bergego Scale

	Catherine Bergego Scale: Neglect			
	
	Baseline	Post-therapy	6-mo Follow up	Change
S1:	5	3	4	-1
S2:	4	3	2	-2
S3:	12	2	3	-9
S4:	16	11	4	-12

Note: Lower score of CBS means a reduction of neglect (0 = normal, 30 = severe neglect).

The different responses of this pilot research could be explained by patients' heterogeneity, as patients differed in terms of age, time post-stroke, affected brain areas, sensation, muscle tone, etc. (Table 4) - all factors that influence motor relearning. The highest motor recoveries were experienced by patient 1, the youngest and least chronic patient. But note that patient 1 like patient 4 received more intensive intervention than the other two patients. It seems that treatment, with additional therapy hours, is primarily fruitful and beneficial for patients with a certain level of remaining sensory functions and motor abilities.

In a comparable study that has been conducted with the robot ARMin I, patients I and II received 24 hours of therapy, while patient III received 40 hours. The improvement of patients I, II and III were +3.1, +3.0, and +4.2 points (initial FMA scores were 14, 26 and 15).

The reason for the less distinctive improvements with the former version ARMin I and other previous robots might be due to the limited movement capabilities (only proximal [14,47,48] or distal [49] arm involvement) or the missing control of proximal joints (endeffector-based robots). In contrast, ARMin II allows for authentic motion sequences, including coordinated interactions between wrist, elbow and shoulder joints. This seems to be an important feature since most everyday activities are composed of inter-joint coordination. ARMin II is an exoskeleton-based robot and, in general, better suited to train ADL-tasks than an endeffector-based robot. This is because, in an exoskeleton robot, the human arm is very well supported and guided by the robot, movements with large ROM can be trained, and the interaction torques that the robot apply to each joint of the human arm can be controlled individually.

Complex movements also enable patients to break abnormal synergy patterns that are limiting arm motor functions [50-52]. As ARMin II provides support against gravity, abnormal synergy patterns in hemiparetic limbs can progressively be learned to be overcome, a matter that was observed in patients 1, 2 and 3. For this therapy issue, the labyrinth scenario seemed to be particularly suitable, as the parameters can be highly varied and adapted individually to the patient's needs. Similar to these findings, Sukal et al. [53] have shown in their research that a reduced range of motion in stroke patients was a result of pathologic synergies during arm lifting. By de-weighting and supporting the impaired arm in training sessions, the effect of gravity could be overcome.

Another approach by Ellis et al. [52] demonstrated that abnormal joint torque coupling could be modified by 'multi-DOF progressive resistance training' in severely impaired chronic stroke individuals. Patients gained strength and simultaneously improved in multi-DOF joint torque combinations. The same relationship could also be observed in the patients that participated in this pilot study. An increase in muscle strength was associated with a larger active range of motion as well as improved muscle coordination. Patients dissociated from synergistic co-activation in the FMA and WMFT.

A distinct finding of the ARMin II study was that post-treatment further progress was achieved in the FMA. These continuing improvements might be due to therapy of distal arm functions since neither the ARMin I study showed such additional effects at follow-up nor any other proximal robotic study in literature that the authors are aware of. Krebs et al. [41] found that training of the more distal limb segments led to twice as much carryover effect to the proximal segments than vice-versa. Moreover, they observed that improvement in more distal segments continued significantly even without further training for that particular limb segment. This finding supports our assumption that the patients were better able to use their arm in daily activities after robotic treatment, allowing them to further improve at the six-month follow up.

With eight weeks of robot training, patient 1 enhanced his performance to such an extent (from initially 21 points to 50 points in the follow-up in the FMA) that he reached a higher functional state - opening up new therapy approaches like constrained induced movement therapy CIMT.

In the present study the two game scenarios (labyrinth and ball game), were particularly suitable to create an enjoyable, efficient and motivating intervention. Patients' interests could be incorporated into therapy by choosing different game settings and levels with miscellaneous arm positions and various joint axes. Nevertheless, complementary therapy modes focusing on specific ADL-tasks and/or virtual reality scenarios might additionally help to facilitate a transfer to ADLs [26,27]. An additional hand module for opening and closing hand function and/or single finger functions would enable more specific and individualized therapy of hand and fingers, allowing for the implementation of more authentic ADL-tasks.

So far, treatment with robotic devices [17,19,54] shows no consistent improvement in functional abilities of daily activities. Although high functional improvements and a transfer to ADLs were achieved in this investigation, these findings are limited to single cases. The pilot study included only four, rather heterogeneous chronic stroke patients. Despite the fact that functional stability could be verified in all patients at baseline, no separate control group was used. All patients continued with their conventional outpatient therapies (maximum 1 hour of physical and occupational therapy per week, focusing on the gait and posture only). However, the patients were encouraged to continue their standard therapies on a constant level, so that possible improvements due to this small amount of conventional therapy can be excluded from the study. Overall, these encouraging results definitely justify starting a large randomized clinical trial.

## Conclusion

This paper presents preliminary results of a pilot study with four heterogeneous, chronic stroke patients using the robotic device ARMin II. The findings support the assumption that robot therapy can significantly influence therapy outcomes. A robot that includes proximal and distal joints, such as ARMin II, allows for a wide field of specific training modalities with natural and complex motions.

In this study it was noted that a subgroup of patients could achieve a transfer to ADLs after performing the training phase. This finding is of great importance since treatment with robotic devices to date did not result in consistent improvement in activities of daily living. A transfer to everyday life should be indeed a central intention of rehabilitation as it brings independence and improve quality of life.

## Competing interests

Tobias Nef and Robert Riener are inventors of patents describing the ARMin invention (WO2006058442, EP1827349, EP07020795). The owners of the patents are the ETH Zurich and the University of Zurich.

## Authors' contributions

PS developed the study design, performed subject recruitment, data acquisition and statistical analysis. She was the primary composer of this manuscript. TN provided feedback and expert guidance throughout this study and was also involved in data analysis. TN and RR designed and built the robotic device ARMin II used in this work. All four authors contributed significantly to the intellectual content of the manuscript and have approved the final version to be published.
